# BK Polyomavirus-Associated Urothelial Carcinoma Presenting as Obstructive Uropathy in a Long-Term Lung Transplant Recipient

**DOI:** 10.7759/cureus.112571

**Published:** 2026-07-13

**Authors:** Austin Iroegbu, Shivam Ghosh, Prabhat Narayan, Sonpreet Rai

**Affiliations:** 1 Urology, Milton Keynes Hospital NHS Foundation Trust, Milton Keynes, GBR; 2 Surgery, Milton Keynes Hospital NHS Foundation Trust, Milton Keynes, GBR

**Keywords:** bk polyomavirus, computerised tomography (ct), obstructive hydronephrosis, obstructive uropathy, post transplant malignancy, urothelial malignancy

## Abstract

A man in his 40s on long-term immunosuppression with bilateral lung transplantation due to cystic fibrosis presented to the hospital with dysuria and flank pain, initially attributed to pyelonephritis. Repeated computed tomography revealed progressive bilateral hydronephrosis unresponsive to ureteric stenting, an extensive bladder wall lesion with calcification, and retroperitoneal lymphadenopathy. A cystoscopic biopsy reviewed at a tertiary centre confirmed high-grade urothelial carcinoma with extensive glandular differentiation, positive for the SV40 antigen, consistent with BK polyomavirus-associated carcinoma (G3 T1 N3 M1a). Persistent hydronephrosis despite appropriately situated stents was the pivotal imaging finding, prompting reassessment for extrinsic malignant obstruction and bilateral nephrostomy insertion. This case highlights the diagnostic challenge of distinguishing malignancy from infection in immunosuppressed individuals through serial imaging.

## Introduction

BK polyomavirus is a pervasive pathogen for which over 75% of the adult population worldwide is seropositive for [1]. Primary infection typically occurs during early childhood, where the virus remains dormant in the renal epithelium but can reactivate under pharmacological immunosuppression, causing nephropathy in transplant patients. Its role as an oncogenic virus is increasingly recognised, with studies identifying aggressive urothelial carcinomas that express SV40 large T antigen almost exclusively in immunosuppressed patients [2]. These tumours characteristically show variant morphology and frequently present at an advanced stage.

The diagnosis of bladder malignancy in transplant recipients is challenging due to the clinical and imaging features overlapping considerably. We present a case in which serial cross-sectional imaging, along with a bladder biopsy, was crucial in navigating the diagnostic pathway from suspected urinary tract infection to rare BK polyomavirus-associated urothelial carcinoma, in which the failure of ureteric stents to relieve hydronephrosis was a key radiological finding that prompted the correct diagnosis.

## Case presentation

A man in his 40s presented to a district general hospital with a several-week history of worsening dysuria and was referred to the same-day emergency care unit (SDEC). He had bilateral sequential single lung transplants for cystic fibrosis approximately 18 years prior and remained on immunosuppression (mycophenolate mofetil, tacrolimus, prednisolone) with prophylactic co-trimoxazole and azithromycin. Relevant past medical history includes cystic fibrosis-associated diabetes, chronic *Pseudomonas* colonisation, and previously excised anal Buschke-Löwenstein tumour (another virus-associated neoplasm arising from immunosuppression). He also had extensive antibiotic allergies.

He reported progressive dysuria and left flank pain, denying fevers or reduced urine output. Examination revealed left loin tenderness but was otherwise unremarkable. He was treated for pyelonephritis with IV gentamicin as an inpatient for five days. Mycophenolate was withheld, and prednisolone was doubled after discussion with the transplant centre.

Approximately 10 days after discharge, he re-presented with worsening lower abdominal pain radiating to the groin, persistent dysuria, and deteriorating renal function (creatinine: 352 µmol/L, estimated glomerular filtration rate (eGFR): 17 mL/min/1.73 m² from a baseline of approximately 82, C-reactive protein: 75 mg/L). He was admitted for intravenous fluid resuscitation.

**Figure 1:**
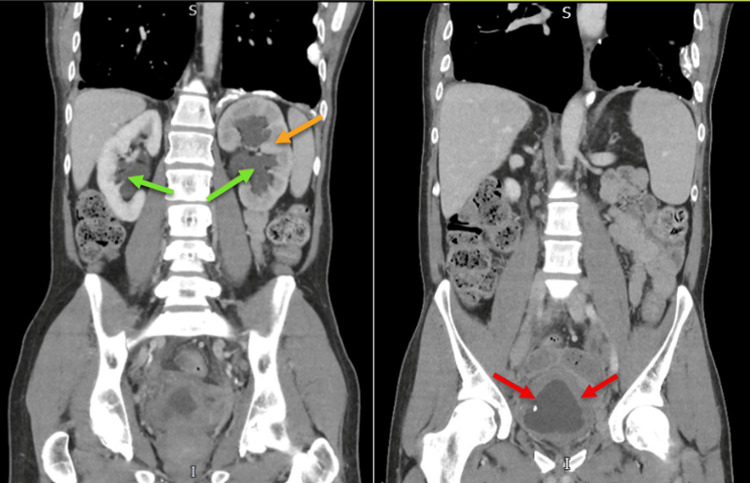
Coronal contrast CT TAP at initial presentation Bilateral hydronephrosis and pelvicalyceal dilatation can be seen (green arrows). The left kidney shows reduced parenchymal enhancement compared to the right, consistent with pyelonephritis (orange arrow). The bladder is visible inferiorly and shows diffuse wall thickening with a 6 mm calcification (red arrows). CT TAP: computed tomography of the thorax, abdomen, and pelvis

**Figure 2:**
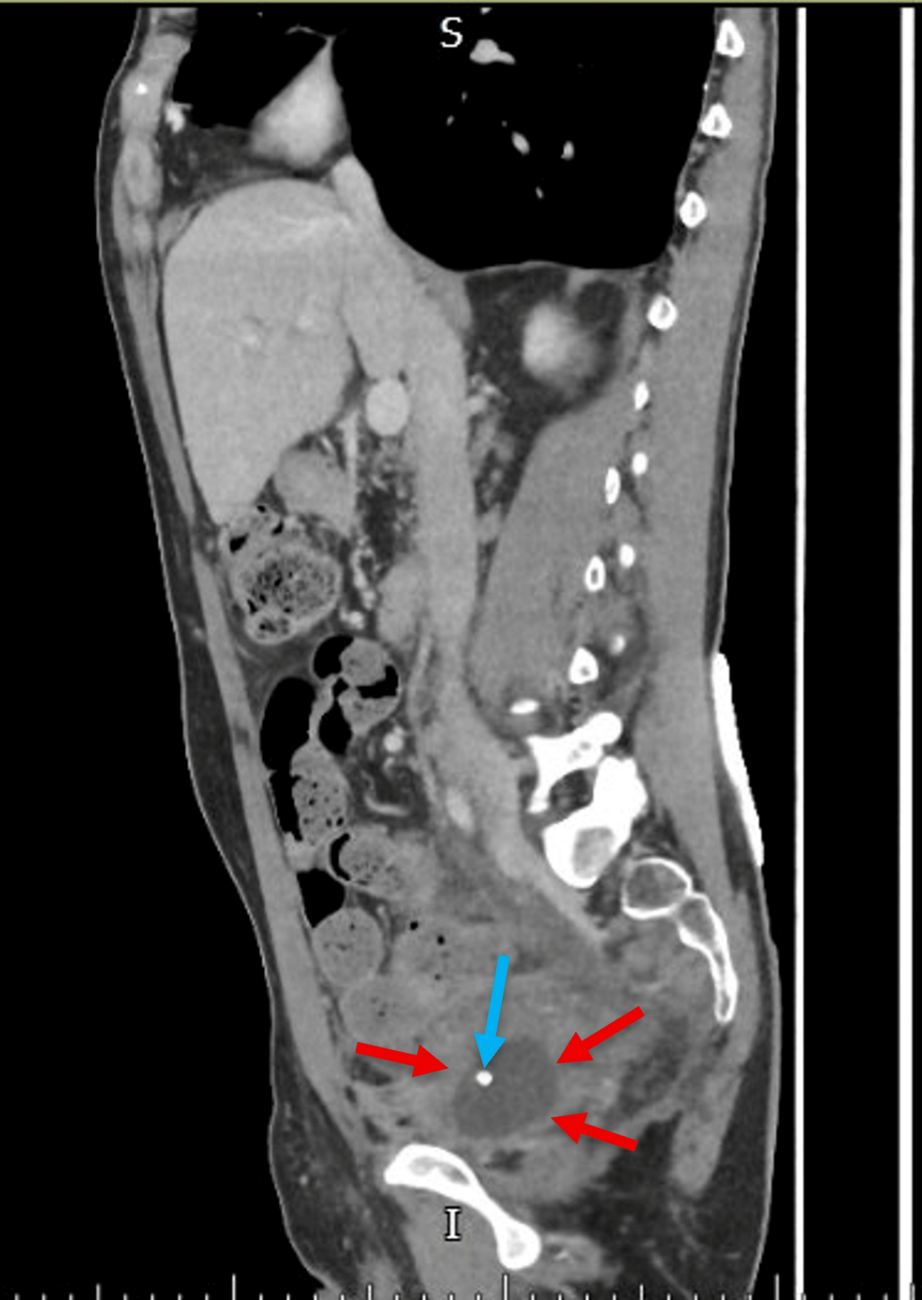
Sagittal contrast CT TAP at initial presentation The urinary bladder demonstrates diffuse wall thickening with a layering enhancement pattern (red arrows). A radiodense focus is identified in the bladder wall, indeterminate for a calculus versus calcification within an underlying lesion (blue arrow). Pericystic fat stranding is present. CT TAP: computed tomography of the thorax, abdomen, and pelvis

Contrast-enhanced CT of the thorax, abdomen, and pelvis at initial presentation demonstrated expected post-transplant changes of the lungs. In the abdomen, the left kidney showed reduced parenchymal enhancement with a dilated, thickened, enhancing left ureter - consistent with pyelonephritis. The right kidney showed mild pelvicalyceal prominence. Bilateral hydronephrosis was present (Figure 1). The urinary bladder was thick-walled and oedematous, with a 6 mm radiodense focus in the right bladder wall that was indeterminate for a calculus versus calcification within a wall lesion (Figure 2). Fat stranding was noted around the bladder in the pelvis. Para-aortic lymph nodes up to 7 mm were described as possibly reactive. The report concluded that an underlying bladder lesion could not be excluded despite the inflammatory appearances and thus recommended cystoscopy. 

A non-contrast CT of the urinary tract was done shortly after, which showed bilateral dilated pelvicalyceal systems and ureters (left being larger), with no calculi and no interval change.

A renal US in light of worsening renal function showed persistent moderate-to-severe bilateral hydronephrosis, with normal-sized kidneys and no calculi.

A urinary catheter was inserted to ensure adequate drainage of his bladder and to ensure that there is no element of high-pressure chronic retention (HPCR) contributing to the aforementioned findings. Despite a catheter left in for >24 hours, a repeat ultrasound showed persistent bilateral hydronephrosis with worsening renal blood parameters.

Cystoscopy was performed, which showed significant bladder wall thickening along with a calcified lesion. Bilateral double J ureteric stents were inserted, and bladder biopsies were taken. At this point, the eGFR had dropped to 9 mL/min/1.73 m².

Post ureteric stent insertion, his eGFR improved to 59 mL/min/1.73 m², and he was discharged with a plan to repeat his kidney function tests in an ambulatory unit and follow up with biopsy results.

**Figure 3:**
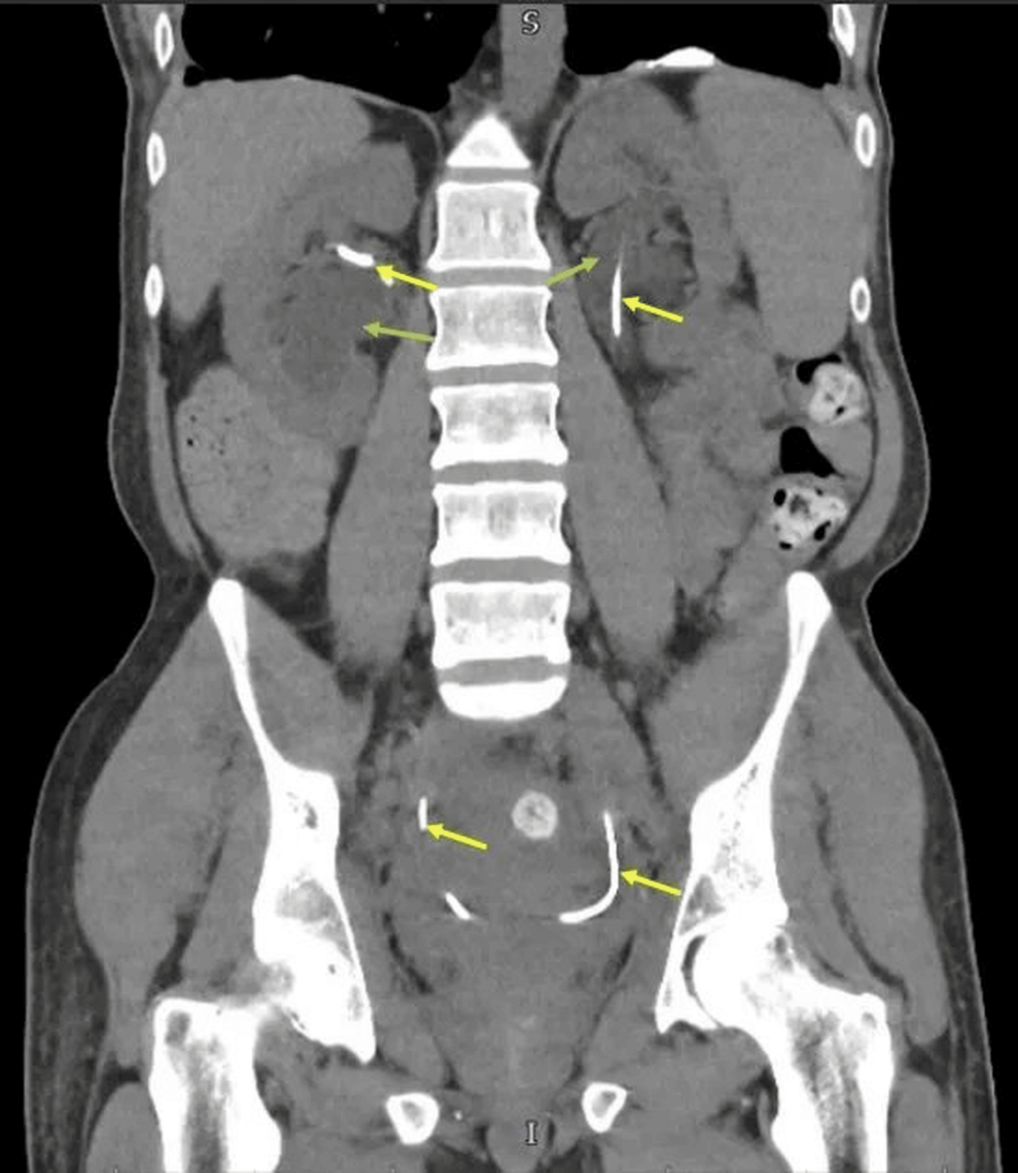
Coronal non-contrast CT of the urinary tract performed post bilateral JJ stent insertion Both stents are seen in satisfactory positions (yellow arrows). Despite appropriately sited stents, bilateral hydronephrosis and pelvicalyceal dilatation persist (green arrows), with slight worsening on the right when compared with previous imaging. This is consistent with extrinsic ureteric obstruction rather than an intrinsic cause that is amenable to stenting.

A subsequent non-contrast CT of the urinary tract, roughly two weeks post stent insertion, was the pivotal study. It demonstrated that the bilateral hydronephrosis persisted and had worsened on the right side despite appropriately situated stents (Figure 3). Extensive soft tissue abnormality within the bladder and retroperitoneal lymphadenopathy were demonstrated. This finding of persistent hydronephrosis despite appropriately sited stents indicated extrinsic ureteric obstruction. His renal function had also dropped to an eGFR of 16 mL/min/1.73 m².

Bladder biopsy showed a tumour with complex architecture, including glandular areas, a villoglandular pattern, and comedonecrosis with calcifications, demonstrating high-grade atypia with lamina propria invasion. Immunohistochemistry showed strong GATA3 positivity and focal CDX2 positivity. Expert review at a tertiary centre reclassified this as a high-grade urothelial carcinoma with extensive glandular differentiation (G3, T1 at least). Importantly, a supplementary report demonstrated strong SV40 antigen positivity (a surrogate marker that cross-reacts with BK Polyomavirus) consistent with BK polyomavirus-associated carcinoma. HPV in-situ hybridisation was negative.

As per advice from the urology multidisciplinary team meeting, a CT thorax with contrast was done and showed no pulmonary metastases or mediastinal lymphadenopathy. Flexible sigmoidoscopy to exclude a colorectal primary source was abandoned as the patient declined bowel preparation. The bladder was accepted as the primary site based on the immunohistochemical profile.

Differential diagnosis

The initial presentation was indicative of a urinary tract infection. As the case progressed, the differential for a thick-walled bladder with calcification and bilateral hydronephrosis in an immunosuppressed patient included severe chronic cystitis, HPCR, primary urothelial carcinoma, primary bladder adenocarcinoma, squamous cell carcinoma, secondary pelvic malignancy, schistosomiasis, and cyclophosphamide-associated cystitis. The initial radiologist's observation that the bladder wall calcification might represent a lesion rather than a calculus was essential to keeping the differentials open. The degree of bilateral hydronephrosis observed on imaging was felt to be disproportionate to the duration of symptomatic dysuria, suggesting the obstruction had likely been developing subclinically over a more prolonged period prior to presentation, consistent with a slowly progressive malignant process. The persistent hydronephrosis despite stent insertion shifted the diagnosis from infection toward malignancy, ultimately confirmed by histology and immunohistochemical profile (GATA3-positive, SV40-positive, HPV-negative).

Treatment

Following the pivotal CT demonstrating stent failure, the multidisciplinary team between urology, nephrology, and interventional radiology concluded that urgent percutaneous decompression was required. Bilateral 8F locking pigtail nephrostomy tubes were inserted. Tacrolimus levels were monitored throughout, with immunosuppression adjusted with advice from the transplant team. Nephrotoxic medications were held.

Outcome and follow-up

This case was discussed at a specialist uro-oncology regional multidisciplinary team meeting, with staging confirmed as G3 T1 N3 M1a (TNM Classification of Malignant Tumours, 8th Edition [3]). The patient was referred to medical oncology for consideration of systemic therapy. At the time of writing, he remains under active oncological follow-up with bilateral nephrostomies in situ with a plan to remove his non-functional bilateral ureteric stents. Renal function stabilised before discharge to 58 mL/min/1.73 m². Table 1 presents the timeline of key clinical events, investigations, and interventions.

**Table 1:** Timeline of key clinical events, investigations, and interventions CT TAP: Computed tomography of the thorax, abdomen, and pelvis; CT KUB: computed tomography of the kidneys, ureters, and bladder; MDT: multidisciplinary team

Timepoint	Clinical Event
Weeks 1-2 before admission	Several week history of worsening dysuria and left flank pain develops. Obstruction likely subclinical and progressive at this stage.
Initial presentation	Referred to same-day emergency care. Examination: left loin tenderness. Treated as pyelonephritis with IV gentamicin (5 days). Mycophenolate withheld; prednisolone doubled in liaison with the transplant centre.
Initial CT TAP (contrast-enhanced)	Bilateral hydronephrosis; left pyelonephritis pattern; thick-walled bladder with 6 mm indeterminate calcified focus; possibly reactive para-aortic nodes up to 7 mm. Cystoscopy recommended.
Non-contrast CT KUB (shortly after CT TAP)	Bilateral dilated pelvicalyceal systems and ureters. No calculi. No interval change. CT urogram and cystoscopy recommended.
10 days post-discharge (re-presentation)	Worsening lower abdominal pain, persistent dysuria, acute kidney injury (creatinine 352 µmol/L, eGFR 17 mL/min/1.73 m²). Admitted for IV fluid resuscitation.
Renal ultrasound (second admission)	Moderate-to-severe bilateral hydronephrosis. Normal renal size. No calculi. Catheter in satisfactory position.
Cystoscopy + bilateral JJ stents + bladder biopsies	Significant bladder wall thickening and a calcified lesion on cystoscopy. Bilateral double-J ureteric stents inserted. Bladder biopsies taken. eGFR at nadir: 9 mL/min/1.73 m².
Post-stent discharge	eGFR improved to 59 mL/min/1.73m². Discharged with a plan for ambulatory renal function review and biopsy follow-up.
2 weeks post-stent (non-contrast CT KUB) - pivotal study	Bilateral hydronephrosis persists; worse on the right despite stents in satisfactory position. Extensive bladder soft tissue abnormality. Retroperitoneal lymphadenopathy. eGFR dropped to 16 mL/min/1.73 m². Extrinsic ureteric obstruction identified.
Bladder biopsy result (local report)	High-grade tumour with villoglandular pattern, comedonecrosis and calcification; lamina propria invasion. GATA3 strongly positive; CDX2 focally positive. Adenocarcinoma queried; referred to tertiary centre.
Tertiary histopathology review (1st report)	Reclassified as high-grade urothelial carcinoma with extensive glandular differentiation (G3, T1 at least). SV40 immunohistochemistry requested.
Tertiary histopathology review (2nd report)	Strong, diffuse SV40 large T-antigen positivity - surrogate marker cross-reactive with BK polyomavirus T-antigen, consistent with BKPyV-associated carcinoma. HPV in-situ hybridisation negative.
Bilateral nephrostomy (interventional radiology)	Bilateral 8F locking pigtail nephrostomy tubes inserted under ultrasound guidance following MDT agreement for urgent percutaneous decompression. No immediate complications.
Flexible sigmoidoscopy (attempted)	Procedure abandoned due to inadequate bowel preparation. Patient declined repeat preparation. Bladder accepted as primary site based on GATA3 and SV40 positivity.
CT thorax (staging)	No pulmonary metastases. No mediastinal, hilar, or axillary lymphadenopathy. No sinister osseous lesions.
Uro-oncology regional MDT meeting	Staging confirmed: G3 T1 N3 M1a (urothelial carcinoma with glandular differentiation, nodal spread, distant nodal metastasis). Referred to medical oncology.
At time of writing	Under active oncological follow-up. Bilateral nephrostomies in situ. Plan to remove non-functional bilateral ureteric stents. Renal function stabilised at 58 mL/min/1.73 m² prior to discharge.

## Discussion

BK polyomavirus-associated urological carcinoma is a rare but increasingly recognised entity. The virus infects 65-90% of adults, remaining latent in the uroepithelium. Under calcineurin inhibitor-based immunosuppression, it can reactivate. The association between this pathogen and nephropathy is well established; however, the oncogenic potential has only recently been characterised.

Sirohi et al. identified SV40 large T-antigen as a reliable immunohistochemical marker with tumours frequently showing variant morphology and presenting at an advanced stage [2]. Roberts et al. highlighted polyomavirus T-antigen expression in post-transplant bladder carcinomas from John Radcliffe Hospital, Oxford [4]. Kenan et al. linked BK polyomavirus genomic integration to oncogenesis [5].

From an imaging perspective, this case highlights two main principles. First, inflammatory bladder wall thickening and pericystic fat stranding can be seen in both infection and malignancy, making the distinction on a single study challenging [6]. The development of findings (persistence and progression despite infection treatment) is what shifted the differential. Second, persistent hydronephrosis, despite appropriately positioned stents (Figure 3), is a recognised indication of extrinsic ureteric compression [7]. In this case, management diverges from re-stenting towards percutaneous nephrostomy, which was the definitive management here.

Transplant recipients carry standardised incidence ratios of 2-5 for most solid tumours and higher for virus-associated cancers [8]. Emerging evidence supports screening for BK polyomavirus colonisation in high-risk populations [9]. No specific treatment guidelines for BK polyomavirus-associated carcinomas currently exist - management is extrapolated from conventional urothelial carcinoma, with consideration of immunosuppression reduction. It is also worth noting that BK polyomavirus is independently recognised as a cause of direct ureteric stenosis through infective ureteritis, historically the first clinical manifestation described for this virus [10]. This represents a distinct mechanism of obstruction from the extrinsic compressive process seen in the present case, and is a differential that should be borne in mind when transplant recipients on immunosuppression present with new ureteric obstruction.

## Conclusions

Persistent or worsening hydronephrosis, despite appropriately positioned ureteric stents, may indicate extrinsic obstruction and should prompt urgent investigation for compressive causes, such as malignancy or lymphadenopathy, with percutaneous nephrostomy as the appropriate decompressive intervention. In immunosuppressed transplant recipients, inflammatory and neoplastic bladder pathology produce overlapping CT appearances; serial imaging and clinical correlation are essential to avoid premature diagnostic closure. BK polyomavirus-associated urothelial carcinoma is a rare, aggressive tumour arising in transplant recipients, characterised by variant morphology and SV40 antigen positivity. It should be considered when bladder wall thickening or a mass is identified in this cohort. Bladder wall calcification on CT that cannot be confidently attributed to a simple calculus should prompt further investigation, including cystoscopy, even when the clinical picture favours infection. A multidisciplinary approach, including team members from nephrology, transplant, radiology, and oncology, is pivotal in such cases.
